# Subaltern housing policies: Accommodating migrant workers in wealthy Geneva

**DOI:** 10.1177/09697764231167091

**Published:** 2023-04-25

**Authors:** Maxime Felder, Luca Pattaroni

**Affiliations:** Erasmus University Rotterdam, The Netherlands; EPFL – Swiss Federal Institute of Technology Lausanne, Switzerland; EPFL – Swiss Federal Institute of Technology Lausanne, Switzerland

**Keywords:** Guestworkers, housing, informality, migration, Switzerland

## Abstract

In the wealthy and orderly city of Geneva, Switzerland, accommodation centres built in haste between the 1950s and the 1980s to house seasonal guestworkers from southern Europe are still standing and still inhabited. Today’s residents are precarious workers, undocumented or with temporary permits as well as asylum seekers. While the seasonal status disappeared in the early 2000s, the demand for low-skilled, flexible labour did not. Analysing the historical trajectories of specific buildings helps us to answer the question of who replaced the seasonal workers, not only in the labour and the housing markets, but also in the symbolic spectrum of legitimacy. This article introduces the notion of ‘Subaltern Housing Policies’ to account for the public action that leads to the production and subsequent use of forms of housing characterised by standards of comfort and security far below those of the rental and social housing stock, but considered ‘good enough’ for their occupants. We argue that ‘subaltern’ relates not only to housing conditions, but also to the policies themselves, and last but not least to the people who are subjected to them. This notion allows us to trace a link between the production of substandard forms of housing and the production of categories of people who are kept on the margins of full citizenship.

## Introduction

It is my opinion that this kind of housing is outdated, but it will take a few more years before we find the right solution, i.e. to build permanent housing specially designed for these workers (Geneva State Councillor, JdG, 14 October 1972).

That was in 1972. This elected official probably could not have imagined that these ‘temporary’, buildings built 11 years earlier to house Italian seasonal workers would still be standing and housing precarious workers 50 years later, in 2022. This article is based on the analysis of three cases of such accommodation centres originally built for seasonal guestworkers between the 1950s and 1980s, and still in use in the early 2020s, housing irregular migrants, precarious migrants with short-term permits and asylum seekers.

First, our historical analysis provides a unique perspective on the transformations of migration and citizenship. Switzerland was a pioneer in the recruitment of foreign labour, developing ‘the most comprehensive migrant rotation system’ (Plewa, 2013) in the second half of the 20th century. While the seasonal status disappeared in the early 2000s, the demand for low-skilled, flexible labour did not. We suggest that analysing the historical trajectories of specific buildings can help answer the question of who replaced the seasonal workers, not only in the labour and the housing markets, but also in the symbolic spectrum of legitimacy.

Second, our analysis focusses on how categories of migrants excluded from the social housing policy nevertheless benefit from some form of public action that provides them with accommodation or at least addresses their housing conditions. Following the work of Spink (2019: 197), our understanding of public action includes the many ‘ways in which the public takes part in defining issues and deciding on actions, alongside and with governments’. We identify patterns of public action in the field of migrant workers’ housing since the end of the Second World War: public authorities act by supporting semi-public or non-profit private actors, through guarantees and also through loans at preferential rates, and by resorting to different forms of service provision by private actors. We will argue that this form of public action can be captured by the notion of ‘Subaltern Housing Policies’ (SHP). Subaltern is considered here as a relational position (Roy, 2011), a position on the margins of the welfare and citizenship system.

The notion of SHP accounts for the public action that leads to the production and subsequent use of forms of housing characterised by standards of comfort and security far below those of the rental and social housing stock, but considered ‘good enough’ for their occupants. It provides analytical ways of making sense of the ambiguous role of the state in the production of informal, or at least substandard, housing, while at the same time allowing us to engage symmetrically with different categories of migrants who are treated separately in policy and research. It links the structurally dominated position of precarious migrant workers with their poor housing conditions.

The city of Geneva is an ideal case study: its housing market has historically been tight, partly due to its geography and the constraints imposed by the lake, the mountains, protected agricultural areas and the French border. While finding housing is a challenge even for well-off citizens, for precarious migrant workers, the challenge seems insurmountable. Nevertheless, the city has been a major entry point for seasonal workers and still has the largest share of both regular and irregular migrants in Switzerland (Federal Statistical Office, 2022; Morlok et al., 2015). Geneva shares this combination of a tight housing market and a large migrant population with many other Western cities, making the case instructive for future comparisons.

We begin with a brief review of the literature on the production of substandard housing and the role of the state in the production of informality. We will then explain the research design and describe the data. The empirical part of the article opens with a history of the rise and fall of the seasonal worker status in Geneva and Switzerland, followed by an examination of three groups of residential buildings built in 1955, 1961 and 1987 for seasonal workers and still in use today. This will provide the ground for our comparative analysis around the conceptualisation of the notion of SHP.

## The housing question of migrant workers

After 1945, several European countries resorted to guestworker programmes to meet their need for low-skilled labour. The aim was to recruit a flexible workforce while limiting immigration: France, the Netherlands, Germany and the United Kingdom, and also Switzerland, issued temporary work permits with few rights and no possibility of family reunification. This system was opposed by the trade unions, while the workers demanded rights, started families and for some settled permanently. Meanwhile, the Western European industries had become dependent on this workforce (Castles, 2006).

While these dynamics led to gradual changes in immigration regimes from the 1970s onwards (especially after the 1973 oil crisis), one key element persisted: migrant workers remained an economically disadvantaged and discriminated category, as reflected in their housing conditions. The post-war period was marked by a housing shortage, but workers’ limited rights did not allow them to obtain housing anyway. In countries, such as Germany and Switzerland, employers were required to provide housing for their employees, although this rule was not always enforced (Miller, 2018). Nevertheless, many guestworkers shared rooms or dormitories in employer-owned accommodation, a situation that, as Madden and Marcuse (2016: 313) point out, was the norm before housing became a commodity. These arrangements also allowed for closer monitoring of workers’ political activism.

In France, workers coming from Portugal, Spain and Italy settled in shanty towns in the 1930s before being joined by Algerians in the post-war period. Between the 1950s and the 1970s, social housing agencies and semi-public organisations built migrant workers’ residences, often run by employers’ associations or charities. These residences had a common characteristic, as Béguin (2011, our translation) points out:

they are built and managed in a framework that derogates from that of the ordinary social housing. This is true in terms of construction, but also in terms of the rights of the occupants, who do not have the status of tenants, since the residences were initially managed as hostels.

Although some residences in France have survived and have been transformed, they have not been fully integrated into the common law system. Their guests are more diverse and are no longer referred to as ‘migrant workers’, but as ‘disadvantaged people’ (Béguin, 2011; Guérin, 2022; Lévy-Vroelant and Mbodj-Pouye, 2022).

This use of selective toleration (Smart and Aguilera, 2020) of non-compliance illustrates the role of the state in the persistence of informal or substandard forms of housing. Some authors see this as a strategic use of informality (Jaffe and Koster, 2019), to meet urgent needs (Picker, 2019). Others point to the consequences of immigration policies that create categories of irregular migrants who cannot access regular or social housing, and also of housing policies that target certain groups and exclude others in the process (Aguilera and Vitale, 2015). Thus, at the core of the idea of SHP is a dynamic relation between categorisation and differential access to (substandard) housing.

Scholars have also questioned the distinction between informality and formality, encouraging analyses of ‘the roles played by ambiguous, contradictory or incomplete policy formulation and implementation across state institutions in creating spheres of informality’ (Clough Marinaro, 2017: 546). The focus should therefore be on differentiation within informality as suggested by Roy (2005), whose argument could be turned on its head to consider ‘differentiation within formality’. Thus, it is not informality but the possibility of producing a formal order that needs to be questioned, especially when considering the ‘inescapable messiness and incoherencies of States institutions and governing instruments’ (Hilbrandt et al., 2017: 950).

Boudreau et al. (2016: 2388) highlight that ‘in certain territorial zones, in certain policy sectors, or at certain scales, the state can be highly formalized, while in others, it may function in highly informal ways’. This ‘uneven formalization’, coupled with contradictions between the different sectors and levels of the state, produces a fragmented urban citizenship. In this respect, the way authorities allocate housing rights, ‘distributing protections unevenly and inconsistently to urban residents’ (Weinstein and Ren, 2009), is key to the production of categories of precarious populations (in the public discourse: ‘the homeless’, ‘the asylum seekers’ and ‘the irregular migrants’) that can be considered as *subaltern* citizens.

Following Spivak (2010) seminal argument, Roy (2011) argues against ontological and topological readings of the subaltern. For her, the subaltern marks the ‘limits of archival and ethnographic recognition’ (Roy, 2011: 231). She thus calls for a more epistemological exploration of the ‘knowledge conditions’ that would allow for the description and reflection of the internal diversity of urban situations that are subsumed under concepts such as slums. The notion of subaltern, as a relational concept, helps us to see beyond the fragmentation of status and forms of citizenship that has occurred since the post-war period. It could help us to consider, for instance, the extent to which irregular migrants or even asylum seekers, can be seen as today’s seasonal guest workers. It opens up the possibility of analysing the historical transformation of the housing conditions of those structurally on the margins of the welfare state.

## Methods and data

**Figure 1.**
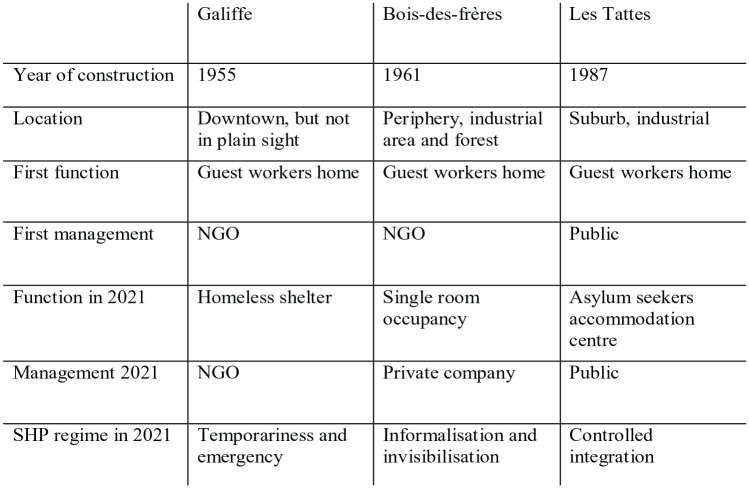
Synthetic presentation of the three cases.

Our study is based on a historical analysis of three cases. We consider a case to be a ‘set of events which has been assembled with the explicit end in view of drawing theoretical conclusions from it’ (Mitchell, 1983: 191). Here, each case consists of the trajectory of a group of buildings from its construction to 2021. We selected the cases not for their typicality, but for their singularity. We chose three groups of buildings built during different decades for seasonal workers and which later took on different functions (Figure 1). Our strategy was to look for patterns in the three historical trajectories that would inform us about the transformations in public action on migrant housing, and to look for possible paths between the seasonal status and other legal statuses.

**Figure 2.**
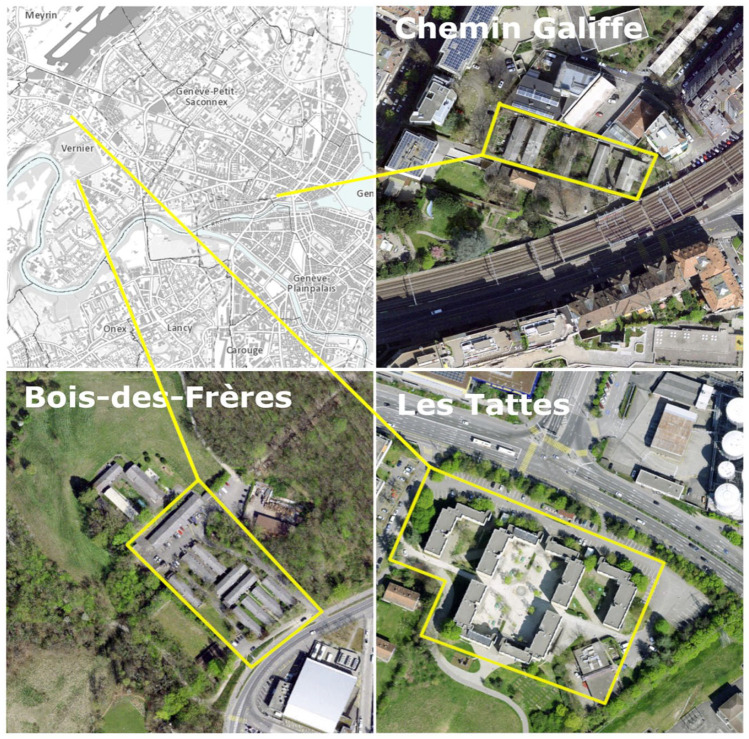
Produced with data from the Système d’information du territoire à Genève (SITG), extracted on the 26 October 2022.

The first group of buildings was built in the city centre in 1955 and has always been run by the Salvation Army. It then became a homeless shelter. The second was built in 1961 and is now a single-room-occupancy accommodation for low-wage workers. It is located between a forest, a river and an industrial zone. The third was built in 1987 and is now a home for asylum seekers. Like the second, it was built in an inner suburb – the city of Vernier – whose population grew massively between the 1960s and the 1970s (Figure 2).

To trace the evolution of the three groups of buildings, we rely on secondary data. First, we have analysed press archives, using a digital library that gathers the archives of *Le Journal de Genève* (JdG, 1826–1998). This daily newspaper was considered as liberal and humanist. Its journalists sometimes expressed sympathy for poorly housed precarious workers, but rarely criticised the authorities and contractors. Critical voices appear in the statements of trade unions and non-governmental organisation (NGO) representatives. For the contemporary period, we draw on articles from the popular daily newspaper *La Tribune de Genève* (TdG), part of a large press group, and the more critical *Le Courrier* (LC), an independent, left-leaning daily.

By analysing the news media, we mainly establish the chronology of events. Although it is not the focus of our analysis, we do take into account the different ways of dealing with ‘public problems’. Between the 1950s and 1970s, certain events, such as demonstrations, seem to have been deliberately ignored by the media. Such events are recorded in the work of historians and in documents produced by NGOs involved in housing struggles. These organisations document their own work from an advocacy perspective. In 2019, they produced an exhibition and a catalogue of archives and testimonies: ‘We, the seasonal workers . . . Geneva 1931–2019’.

Finally, we use interviews conducted in 2019 and 2020 with the manager of the Bois-des-Frères housing complex, the person in charge of housing asylum seekers and refugees, a Salvation Army social worker, and the director of the NGO ‘Protestant Social Center’. These people will not necessarily be quoted, but they helped us to understand the history and functioning of the different accommodation structures. In the course of our research^1^, we also met with precarious workers, two of whom are quoted in the empirical section, to put flesh on the bones of the migrant workers we are dealing with in this article.

## The rise and fall of the seasonal worker status in Geneva and Switzerland

Since the end of the Second World War, labour migration in Switzerland has been regulated by agreements designed to facilitate the admission of workers while preventing them from settling permanently. The status of seasonal workers, institutionalised by the first federal law on foreigners’ residence in 1931, was intended to ensure sufficient flexibility to meet the needs of the economy while combatting ‘foreign overpopulation’. The authorities relied on the so-called ‘selected immigration’ to provide manpower for sectors subject to strong seasonal fluctuations: construction, agriculture and hospitality industry. Agreements were signed with Italy in 1948 and with Spain in 1961. After the oil crises of the 1970s, Switzerland relied more heavily on Portuguese emigration to meet its needs for workers.

Seasonal workers (90% of whom were men) left their country for 9 months a year to work in Switzerland, under harsh conditions and with many accidents (LC, 12 July 1973). On arrival, seasonal workers were subjected to humiliating health checks, during which those deemed unfit to work were repelled, suggesting their reduction to ‘productive bodies’ (Santos Rodriguez, 2022: 98). Their housing conditions were often described as indecent and their rents exorbitant. Family reunification was forbidden, which led to the birth of thousands of undocumented children between 1960 and 1980 (Ricciardi, 2010).

Seasonal workers could not change jobs nor move to another canton without permission. If they lost their job, they had to leave the country. This allowed Switzerland to ‘export unemployment’ in the 1970s (Afonso, 2005: 658). Seasonal workers were part of ‘a foreign workforce whose manipulation made it possible to absorb virtually all of the costs of change in the international trade system’ (Katzenstein, 1985: 59).

At the end of the 1980s, associations and trade unions fought for the abolition of the status of seasonal workers. The Swiss government’s plan to join the European Economic Area (EEA) provided a favourable context for such a change, but was defeated by popular vote (by 50.3%) in 1992. Change finally came with the agreement on the free movement of persons with the EU in 2002. Industry that depended on seasonal workers began to look outside Europe for substitutes (Afonso, 2005: 663). However, the federal authorities and the right-wing parties succeeded in strictly limiting extra-European immigration to the most skilled workers, furthering the idea of ‘selected immigration’.

In summary, the number of seasonal workers rose sharply in the 1950s and 1960s, before falling temporarily after the 1973 crisis and then permanently in the 1990s, until the abolition of this status in 2002.

## Flexible housing for a flexible workforce

The housing of seasonal workers has been a matter of debate and concern from the outset. These workers were imported to solve the (social) housing shortage, but not only did they not have access to social housing, but their legal status also prevented them from signing a lease. While the 1948 agreement with Italy required employers to provide accommodation (deducted from wages), the subsequent agreement with Spain ensured that the workers would ‘enjoy the same rights and protection as nationals with regard to the application of laws on health and work, as well as housing’. But the reality was very different.

In the mid-1950s, the local press began to report on the housing conditions of the workers. Many were homeless, living in shelters and sometimes sleeping rough. As a result of the publicisation of these poor housing conditions, the laissez-faire approach gave way to a series of original public prescriptions outlining the first elements of what we call SHP. In line with the principle of subsidiarity, local authorities provided interest-free loans to NGOs for the construction of temporary housing. To understand how SHP have developed and evolved with the disappearance of the seasonal status, we will now examine the history of three housing sites built for seasonal workers.

### Chemin Galiffe – 1955

In 1955, with a loan from the Canton of Geneva, the Salvation Army inaugurated three wooden barracks on the Chemin Galiffe, less than a kilometre from the train station. The 100 workers cooked on alcohol stoves in the dormitories. The following year, to avoid an accident and because the beds were still scarce, three new prefabricated barracks were erected, one of which served as a canteen where breakfast and dinner were served ‘at a very reasonable price’ (JdG, 9 December 1956).

During the Hungarian Revolution of 1956, refugees were temporarily accommodated, as many workers had already returned to Italy for the winter. While the Italian workers were treated with some suspicion – out of fear of communism – the Hungarian refugees were presented in the press as ‘the most sympathetic young men full of good will’ (JdG, 27 December 1956). The prevailing anti-communism and their victim status earned them a favourable attitude (Parini, 1997: 56). The press articles emphasised that this solution had to be temporary, as the refugees deserved better housing conditions (JdG, 27 November 1956).

When it came to seasonal workers, however, the journalists judged the level of comfort by different standards, describing the barracks as ‘comfortable’, and praising those ‘who undertake to accommodate, as adequately as possible, the foreign labour force that our canton needs’ (JdG, 30 August 1961). In the same article, however, the journalist mentions a conflict about the poor maintenance, the limited opening hours of the canteen and the fines imposed on the workers for breaking the house rules.

These barracks were also used as shelters for the homeless during the winter, until the seasonal workers returned. The homeless then went back to the streets or were moved, for example, to former military barracks (JdG, 27 February 1960). As the number of seasonal workers decreased, the homeless shelter service became gradually more important. These ‘temporary’ buildings were already 30 years old when an article in 1985 reported that they would only be used as emergency shelters for the homeless. The article stated that they would be demolished within 2 years to make way for sports facilities. In 2021, however, they were still being used as a Salvation Army homeless shelter.

People stayed there for a maximum of ten nights every 3 months, paying five francs a night. According to a social worker we interviewed, many of them are migrants, ‘trying their luck’ in Geneva. We met one of them at the train station. Mihai is a 35-year-old Romanian. Illustrative of circular migration, his journey took him from Spain to Switzerland via France, before temporarily returning to Romania. His plan was to work in the apple harvest, as he had done in previous years. When he earns some money, he will move into the Chemin Galiffe shelter. Until then, he sleeps in a park behind the train station. Other newcomers we met said they had slept at Chemin Galiffe, like Amadou, from Cameroon, who eventually got a temporary job harvesting grapes. All of them were precarious migrant workers who moved regularly between different forms of substandard housing.

Although the buildings were partially renovated, the Salvation Army acknowledged in 2021 that they no longer met safety standards. The buildings were vacated, 66 years after their construction, and a new shelter now accommodates about 60 homeless people in another neighbourhood. Its construction was financed by private donations, the operational costs are subsidised by the city of Geneva and the management remains in the hands of the Salvation Army. In 2022, two associations took over the old barracks to provide housing for homeless women and lesbian, gay, bisexual and transgender (LGBT) asylum seekers.

### Bois-des-Frères – 1961

In the late 1950s, beds in the workers’ dormitories were scarce or considered too expensive, leading some seasonal workers to sleep rough, sometimes directly on the construction sites. The situation was ‘known and tolerated by the authorities’ (JdG, 21 November 1962). Under pressure from NGOs, the government decided in 1960 to reopen abandoned military barracks to be run by the Salvation Army. These were demolished the following year, and 300 workers were relocated to an exhibition centre and to empty industrial premises, with the help of the Protestant Social Centre.

The position of the trade unions changed at this time. While a major construction union protested against the arrival of seasonal workers in the spring of 1959, 2 years later, the same union organised a demonstration to denounce their housing conditions (Steinauer, n.d.). An archive photo shows placards reading: ‘We Italian workers demand: a bed, a table, a chair – is that too much to ask?’ The situation was critical, as Geneva was preparing to welcome 7000 seasonal workers, twice as many as the previous year. The authorities decided to support the construction of 30 wooden barracks, some of them in Bois-des-Frères. This piece of public land was just a stone’s throw from the construction site of what remains the largest housing development in the region: Le Lignon (2780 dwellings), located on the outskirts of the city (JdG, 25 February 1961).

**Figure 3.**
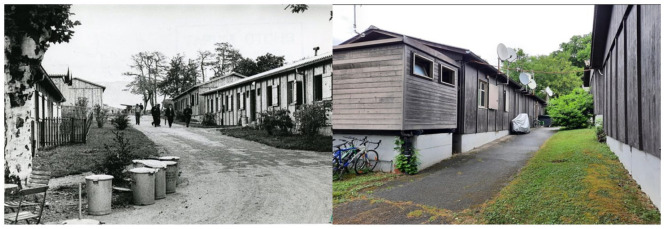
Bois-des-Frères, 1963 (Christian Murat, © Bibliothèque de Genève), 2022 (Authors).

Twelve wooden barracks were built (Figure 3), with 250 beds in small four-bed rooms. The authorities entrusted the Protestant Social Centre with the management of the site. In the autumn of 1968, refugees from the Prague Spring moved in. Once again, the press called on the population to welcome these refugees for whom Bois-des-Frères was not a suitable place to live (JdG, 13 September 1968).

Three years later, when the authorities decided to raise the rents, 300 workers were living there. In a characteristic moment of reconfiguration of SHP, this provoked various reactions: mobilisations, denunciations and also legitimisation of these housing conditions. The workers stopped paying their rent until the promised improvements were made. The NGO gave up running the centre in protest. Nevertheless, a journalist reporting on this event noted how well the seasonal workers were treated: the food was ‘excellent’, the rooms were cleaned and ‘a small recreation room with television, newspapers and books allows the seasonal workers to relax in the most pleasant atmosphere’ (JdG, 14 October 1972). The prohibition on cooking – workers were forced to eat in a paid canteen – or having a radio in the room, the lack of space and privacy did not seem to cause concern.

In the same article, however, the cantonal minister admits that the building is ‘outdated’ (see the opening quote in the ‘Introduction’ section). Although he would like to build proper housing, finding a suitable location is hard: ‘People don’t want seasonal workers in their neighbourhood, it’s sad’ (JdG, 14 October 1972). After the NGO left, the site was taken over by the ‘Group of young business leaders’ who turned it into a private company (JdG, 18 October 1972).

Soon after, as a result of the 1973 oil crisis, the number of seasonal workers declined and the barracks became home not only to seasonal workers but also to other people looking for cheap accommodation, and to refugees (during the Yugoslav wars). From then on, the buildings were only partially occupied. Two of the barracks burned down, in two separate fires, one of which killed a worker. Ten barracks remain, with 135 individual rooms of ten square metres each, reserved for men. As was the case in the 1960s, toilets, showers and kitchens are shared.

After decades of low occupancy, all the beds are now occupied, mostly by Portuguese men. Some are retired, others are low-income workers with L permits, which allow EU citizens to work in Switzerland for short periods. The residents pay their rent – 420 francs – in cash. Despite the low level of comfort, these prices are attractive. ‘I used to accept anyone’, the manager told us, ‘but now our company requires a residence permit, and not too much debt. The premises are certainly outdated, but the people are there by choice’, he insisted. ‘And they stay, the rate of turnover rate is very low’. To our knowledge, this accommodation is not advertised anywhere. According to the manager, people find it by word of mouth.

The managing company remains the same as that which took over from the NGO in 1972. It is still chaired by the founder, a business man and liberal politician who has since been a member of the city government and of the canton’s government.

### Les Tattes – 1987

In the spring of 1970, hundreds of Spanish workers demonstrated. Two weeks later, they went on strike and blocked a large construction site in protest at their living conditions. They were supported by organisations, such as the Association of Spanish Workers in Switzerland (ATEES) and the Federation of the Italian Free Colonies (FCLIs), both close to communist parties. Workers were also supported by far-left political groups (Magnin et al., 2019), who demanded the right to decent housing. In response, habitability standards were introduced and two housing inspectors were hired, further formalising the regulation bundle of SHP. A major step was taken in the late 1980s with the first permanent collective accommodation centre for seasonal workers.

Les Tattes is the result of collaboration between the local authorities, employers and trade unions. Opened in 1987, it consists of 12 buildings arranged around a courtyard and was designed to accommodate 600 people. It was built in a short time, using prefabricated elements. However, the number of seasonal workers dwindled rapidly and by 1993, only about 20 residents remained. The following year, the centre was rented to the Federal Office for Refugees to house asylum seekers.

The buildings were redesigned, as asylum seekers were expected to prepare their own food. Kitchens were installed at the end of each corridor, the old plumbing was replaced, reception desks and office were created, and about 100 rooms were removed. Over time, the centre mainly housed asylum seekers whose applications had been rejected.

In 2014, at the height of the ‘refugee crisis’, the capacity of the rooms was doubled, but the number of bathrooms remained the same, leading to hygiene problems. In the same year, a fire (the third in 4 years) killed one asylum seeker and injured 40 others. In 2023, after a lengthy trial, the court cleared the authorities but convicted two security guards and the resident whose hotplate was believed to have caused the fire. Lawyers for the victims unsuccessfully cited a report highlighting breaches of safety standards to argue that the state was responsible for the building’s poor condition and overcrowding.

Today, the living conditions remain precarious. Rooms are small, lack privacy and are not soundproofed. Strict rules ensure order, but curtail hospitality. Residents are not allowed to invite guests, drink alcohol or bring in personal furniture.

## Subaltern housing policies

Documenting the history of the buildings and their successive occupants over several decades allows us not only to uncover patterns in public action, but also to trace a link between the production of substandard forms of housing and the production of categories of people who are kept on the margins of full citizenship. Both processes relate to what we call here SHP. They link the question of informalisation with that of structural and symbolic domination. We will now explain how ‘subaltern’ relates to housing conditions, and also to the policies themselves, and last but not least to the people who are subjected to them.

### ‘Good enough’ housing conditions

SHP involve the direct or indirect provision of forms of housing that is far below the standards experienced by the vast majority of the resident population. Subaltern therefore refers to a relative rather than an absolute standard.

Although the three groups of buildings we have described have been renovated and improved overtime, at the cost of struggle and mobilisation, they have always remained inferior in terms of comfort and habitability to what was the norm for citizens in their own right. Indeed, they offer few opportunities for appropriation, little privacy and impose promiscuity. Seasonal workers could not offer hospitality to friends and family, could not furnish their rooms, and could not cook for themselves. In 2021, habitability varied from case to case, but remained limited, not least because of the small size of the rooms and the rules imposed on the occupants. The restrictions on the use of the accommodation, but above all the lack of choice, severely limit autonomy and contribute to the subalternisation of residents.

Security of tenure is also a key difference from ordinary housing, which governed by Swiss tenancy law. As in the case of migrant workers’ hostels analysed by Béguin and Guérin, residents are not considered as tenants. While the residents of Bois-des-Frères have a monthly lease and stay for many years, the residents of Chemin Galiffe are usually allowed to stay for a maximum of 10 days at a time and can lose their place if they do not show up on time in the evening.

These conditions cannot be justified by urgency, as in the case of the temporary shelters built for earthquake victims. They result from a lack of foresight and willingness to offer more to these populations. Subaltern, then, refers to the fact that these dwellings were and still are considered ‘good enough’ for their inhabitants. We saw that elected officials and journalists judged the housing conditions much more harshly when the residents were not Italian seasonal workers but refugees fleeing a communist regime.

### Subaltern policies

The institutional arrangements behind SHP are themselves subaltern in the sense that they involve exceptions or partial enforcement of the ordinary regulations. This has to do with their supposedly temporary character. In the first two cases, the buildings were intended to last only a few years. In the third case, the building is permanent, but was designed for temporary stays. The ‘state of exception’ that characterises SHP, implying a regulation of a lesser scope and validity – that is, a lesser ‘degree of formalisation’^2^ – is not the result of the state imposing its sovereignty, but rather the consequence of different state bodies and NGOs having different forms of influence, power and responsibility over the situation (Picker, 2019). Subaltern policies thus denotes a form of public action more than a coherent policy. As Aguilera and Vitale (2015: 71 our translation) argue about the production of European slums: ‘There is no such thing as a slum policy in Europe’. Slum rather results from ‘a set of networks of instruments, practices and discourses, more or less institutionalised, which are superimposed at various levels of government and in a discontinuous manner over time’.

This leads to contradictions and incoherences between different policy elements. As is often the case, these contradictions only become apparent after tragic events (Oppenchaim and Le Mener, 2013), such as the fatal fires that occurred in two of the sites studied. SHP is thus an umbrella concept that allows us to pay attention to how subaltern housing conditions become a public problem. It points to the more or less coordinated action of public, private and civil society actors at the intersection of welfare, asylum, solidarity, charity and market policies.

On one hand, the expansion of SHP reflects the fact that in the course of the 20th century, in accordance with the principle of subsidiarity, the Swiss authorities have increasingly acted by providing incentive, pushing and guiding the third sector into action (Butschi and Cattacin, 1993). On the other hand, the fact that public authorities wait until the situation becomes critical before acting suggests that the purpose of this public action lacks political legitimacy, or rather that the subjects of this public action are not fully legitimate. This could explain why state actors act in an indirect, provisional or even exceptional way.

### Subaltern workers

In the three groups of buildings, seasonal workers rubbed shoulders with other legal or administrative categories before being completely replaced. People categorised as ‘homeless’ were housed in the first two sets of buildings. In Geneva, this category includes a large proportion of irregular migrants and others with short-term permits (Bonvin et al., 2021). Among them, the so-called ‘circular’ migrants – often from Eastern Europe – emerge more clearly as the ‘new’ seasonal workers, responding to the temporary needs of the economy (especially agriculture). For Plewa (2013: 101), ‘circular migration [is] the dernier cri in European migration policy aimed to strike a compromise between a perceived post-crisis demand for the admission of foreign workers and the reluctance to make them prospective citizens’.

Asylum seekers and refugees – who have been housed at least occasionally in all three locations – can also be seen as the new seasonal workers. During the Yugoslav wars, the number of employed asylum seekers in Switzerland exceeded the number of seasonal workers (Piguet and Wimmer, 2000). The abolition of the seasonal status in Switzerland reinforced this trend, giving ‘low-skilled refugees good opportunities to find employment, especially in the restaurant industry’ (Ruedin et al., 2020: 24). Today, asylum seekers constitute one of the most flexible legal workforces and some employers consider them indispensable (Fibbi and Dahinden, 2004).

While the seasonal workers were rather homogeneous groups in terms of origin, gender, age and legal status, the ‘new’ seasonal workers are thus more heterogeneous. This diversification has several consequences. Having the same status and often sharing the same nationality and mother tongue made it easier for seasonal workers to act collectively. As an identified and visibilised public problem (Lichterman, 2021), they received support from NGOs and trade unions, they formed associations, and were defended by the diplomatic authorities of their countries of origin. In 1966, Italian diplomats visited the workers at Bois-des-Frères (JdG, 26 October 1966). The legal status of seasonal workers, although precarious, provided legitimacy to demand rights: not only were they legally in Switzerland, but also they had been ‘invited’. Strikes and demonstrations enabled them to obtain slightly better conditions, but only after decades of struggle. Their final demand – the abolition of the status of seasonal workers – was finally met at the turn of the 21st century.

The disappearance of the seasonal status has improved the lot of workers from Schengen countries, but it has made the others more precarious and invisible. The number of people depended on food aid during the COVID-19 pandemic brought these shadow workers who wash the dishes in restaurants, care for children and the elderly, clean houses and offices, into the limelight (Stavo-Debauge et al., 2022). Like the seasonal workers who were the first to be made redundant after the 1973 oil crisis, these workers were the first to suffer from the health crisis.

SHP stem from a lack of respect for these categories of workers. The contribution of post-war seasonal workers to the economic development of Switzerland is better understood today, but it has never been taken for granted. Suspicions of social dumping have always weighed on migrant workers, and some political parties continue to claim that these jobs could be filled by unemployed locals.

## Three regimes of SHP

The development of the modern state has created a fundamental contradiction between the Kantian ideal of equal human dignity and the selection inherent in the development of a welfare system. The housing question exacerbates this contradiction because it is a particularly scarce resource. Although migration policies can be adapted to the needs of the economy, housing policies can hardly react in the same way. Only a stock of readily available housing would be able to respond to the fluctuating arrival of newcomers. However, it would be absurd to leave dwellings empty in the face of a persistent housing shortage. Even residents who are entitled to social housing are often left waiting.

SHP offer a way out of this dilemma. In the absence of a housing reserve, local actors use a slowly sedimented ‘spatial reserve’: disused land and buildings that can be converted into temporary accommodation. This spatial reserve has been built up over the years with the deindustrialisation of urban centres and the closure and relocation of military barracks, depots, exhibitions and fairgrounds, and so on. The use of churches as temporary emergency shelters for the homeless since 2019 illustrates the renewal and expansion of this spatial reserve, as well as the latest developments of SHP in Geneva. The local (and voting) population is less likely to claim these temporary and uncomfortable accommodation, especially when they are managed by charities, such as the Salvation Army, whose very name evokes poverty.

Our comparison shows the breakdown of SHP into at least three distinct regimes (see Figure 1). The first, illustrated by the case of Chemin Galiffe, is a regime of emergency and temporariness aimed at dealing with poverty (Gardella, 2016). The actors involved in this regime are NGOs active in homelessness relief, but also municipal social services. The types of spatial reserves they activate are, in addition to the seasonal workers’ barracks, nuclear shelters and churches.

The second regime, illustrated by the case of Les Tattes, involves controlled integration. It is based on refugee rights, which legitimise the selection process of those who will have access to integration policies. Residents are housed directly by the authorities and are subject to the criteria and decisions of the asylum authorities. This regime includes accommodation centres for rejected asylum seekers and, in other contexts, refugee camps. It involves NGOs working in the field of asylum, quasi-public bodies responsible for the reception of asylum seekers and national authorities.

The third regime, illustrated by Bois-des-Frères, is characterised by invisibilisation and informalisation. The authorities – who own the land – tolerate these substandard but discreet housing conditions, whose existence is known by word of mouth. This regime relates to what has been known in France since the 1970s as ‘de facto social housing’ (*parc social de fait*), that is, low-cost, low-quality housing that attracts households excluded from social housing. It includes housing run by ‘slum landlords’, who rent rooms and sometimes just mattresses rented at inflated prices, taking advantage of precarious migrants and filling a gap in the housing market (Aalbers, 2006). In Geneva, an association founded in 2020 helps irregular migrants to assert their rights as tenants. This initiative, which originated from the largest Swiss tenant’s association, illustrates how historical housing rights movements are shaping SHP. This regime also includes different squatting situations, as a housing solution for precarious migrants (Dadusc et al., 2019) and as a site for mobilisation (Mudu and Chattopadhyay, 2016). In Geneva, the large-scale political squatter’s scene of the 1980s and 1990s allowed many undocumented migrants to stay in the city and build relationships with local activists.

These three regimes show that public action on migrant workers is now fragmented into different sectors, with different legal statuses. These confer different rights, but still a partial and marginal citizenship. By treating these different statuses symmetrically, as we do in this comparison, we can better identify the effects of this sectorisation and categorisation on housing conditions, and more broadly on the living conditions and life chances of newcomers. Indeed, although we argue that all are subject to SHP, their experiences and their prospects vary enormously, particularly according to their legal status.

## Concluding remarks

On a theoretical level, our analysis reflects the entanglement of two understandings of the notion of subaltern: (1) a subordinate political and spatial condition and (2) a valuation process.

1. The subordinate political and spatial condition does not necessarily entail a marginal location in the city, but it does imply a shared experience of scarce housing space and of specific regulations that limit the duration of residents’ stay, their daily rhythms and routines and their activities (cooking, inviting people). Residents are always prevented from appropriating their housing and thus from fully dwelling. This condition undermines the status of people who are already disadvantaged, both in the labour market and in terms of the legitimacy of their presence on the national territory.
2. Nevertheless, the legitimacy of such a condition is the subject of political controversies. We have seen that the same housing conditions are considered suitable for seasonal workers while appearing inappropriate for refugees from Hungary and Czechoslovakia. In 2022, while the housing conditions of asylum seekers were of little concern, the arrival of Ukrainian refugees raised controversies about their housing conditions. Subaltern thus necessarily refers to a dynamic process of (de)valuation and (de)legitimisation.

The notion of SHP provides analytical avenues for understanding ‘the political-institutional production of migrants’ housing precarity’ (Dotsey and Chiodelli, 2021: 733), opening up possible comparisons between historical and contextual public action regarding the housing conditions of politically and legally distinct populations on the margins of the welfare state.

The intensification and diversification of SHP reveal the crisis of welfare state integration policies, which are confronted with increasingly heterogeneous forms of migration, far from the linear models of the irreversible exile of the 20th century. This complexity is likely to increase with the climate crisis, bringing urban and housing questions closer together in the Global South and North. Reclaiming the concept of ‘subaltern’ in the study of a European city like Geneva is, therefore, a way of opening up avenues for future comparisons with other situations in the Global North, and also in the Global South. Indeed, the figure of the slum needs to be embedded in a discussion around the development of SHP. Our article suggests that in today’s European cities, ‘slums’ can take a more diffuse and less visible form, making it difficult to build up transversal mobilisations among contemporary subordinated populations. The SHP model, as a transversal perspective on precarious migrant workers, is a way of dealing analytically, and also politically, with the fragmentation of claims (Blokland et al., 2015) and a way of resisting the sectoral logic of migration and poverty management.

## Notes

1. ‘(In)hospitalités urbaines: Quelle place pour l’arrivant-e en situation précaire dans les villes de Genève et Bruxelles ?’ Swiss National Science Foundation, grant 182295.

2. We refer here to Laurent Thévenot’s conceptualisation of how ‘investments in forms’ are necessary to formalise and extend the validity of norms and rules (Thévenot, 1984).
